# Thyroid Carcinoma on the Side of the Absent Lobe in a Patient with Thyroid Hemiagenesis

**DOI:** 10.1155/2017/4592783

**Published:** 2017-11-27

**Authors:** Hiroki Sato, Kiyoaki Tsukahara, Ray Motohashi, Midori Wakiya, Hiromi Serizawa, Atsushi Kurata

**Affiliations:** ^1^Department of Otorhinolaryngology, Head and Neck Surgery, Tokyo Medical University, Tokyo, Japan; ^2^Department of Pathology, Tokyo Medical University Hachioji Medical Center, Tokyo, Japan; ^3^Department of Molecular Pathology, Tokyo Medical University, Tokyo, Japan

## Abstract

**Background:**

Thyroid carcinoma complicated by hemiagenesis is very rare, and previous reports have not described this cancer on the side of the absent lobe.

**Methods and Results:**

We report the case of a 64-year-old woman in whom left thyroid hemiagenesis was discovered incidentally during investigations of abnormal sensation during swallowing. A tumorous 1.4 cm lesion was also found on the side of the absent lobe, left of the isthmus. Fine-needle aspiration biopsy revealed class V papillary carcinoma, but no lymph node metastases. Total thyroidectomy was performed for stage cT1bN0M0 carcinoma. Histopathology revealed normal thyroid tissues in the right lobe and isthmus, while the left lobe was absent. The mostly papillary carcinoma was adjacent to the truncated thyroid tissue, with a portion histologically consistent with poorly differentiated carcinoma.

**Conclusions:**

All previously reported cases of thyroid cancer complicated by hemiagenesis have represented carcinoma occurring within the present lobe. This case is extremely rare.

## 1. Introduction

Thyroid hemiagenesis is an extremely rare congenital abnormality, with a reported prevalence of 0.02–0.2% [1, 2]. This pathology is often discovered during detailed examinations for abnormal thyroid function and thyroid diseases such as goiter. Only 13 previous cases of thyroid cancer concurrent with thyroid hemiagenesis have been reported. Tumors in each of those cases occurred in the remaining thyroid tissue; no previous reports have described thyroid carcinoma occurring on the side of the absent thyroid lobe. We report a case of thyroid carcinoma arising on the side of the absent lobe. To the best of our knowledge, this represents the first report of its kind. Although the cause of onset is unclear, we discuss our hypotheses regarding the tumorigenic mechanisms of thyroid carcinoma in this case.

## 2. Case Presentation

**Figure 1:**
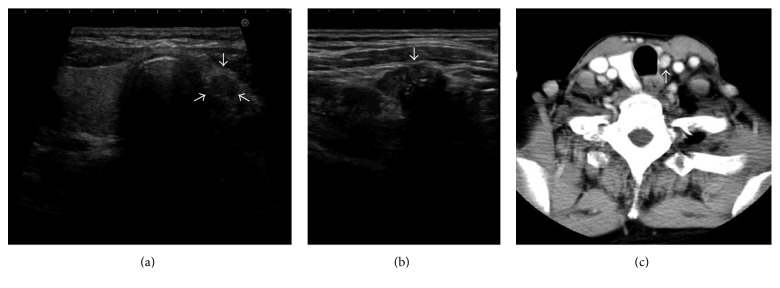
Findings from ultrasonography and computed tomography. The right thyroid lobe and thyroid isthmus are present, while the left lobe of the thyroid is absent. A nodule measuring 0.7 × 0.8 × 1.4 cm is seen on the left side of the thyroid isthmus. On cervical ultrasonography, the periphery of the tumor is nonuniform, small calcifications are detected in the interior, and the posterior echo is attenuated. Arrows indicate the tumor. No cervical lymph node swelling is detected.

A 64-year-old woman was referred to our department for evaluation of a thyroid mass discovered incidentally during examinations for symptoms of abnormal sensation during swallowing. The patient had no history of previous surgery or radiotherapy to the neck. She had no significant past medical history. No family history of thyroid disorders was elicited. Examination of the neck revealed a firm mass with irregular edges and little mobility. No cervical lymphadenopathy was evident. Examination of the oral cavity, pharynx, and larynx showed findings within normal limits, with mobile vocal cords. Ultrasonography and computed tomography (CT) did not show a left lobe of the thyroid, but the right lobe and isthmus were identified. A 0.7 × 0.8 × 1.4 cm hypoechoic nodule with small calcifications and attenuation of posterior echo and a 0.8 × 0.7 cm enhanced nodule with clear borders were identified on the left side of the isthmus (Figure 1). Ultrasonography and CT did not reveal enlarged lymph nodes on either side of the neck. CT also did not reveal metastatic tumors in the lungs. Serum thyroid-stimulating hormone (TSH) was 1.35 μIU/mL (normal range: 0.35–4.94 μIU/mL), free triiodothyronine (FT3) was 2.87 pg/mL (normal range: 1.71–3.71 pg/mL), free thyroxine (FT4) was 1.43 ng/dL (normal range: 0.70–1.48 ng/dL), and thyroglobulin was 12.93 ng/mL (normal range: 0–32.7 ng/mL). Ultrasonography-guided fine-needle aspiration cytology identified the nodule as papillary thyroid carcinoma.

**Figure 2:**
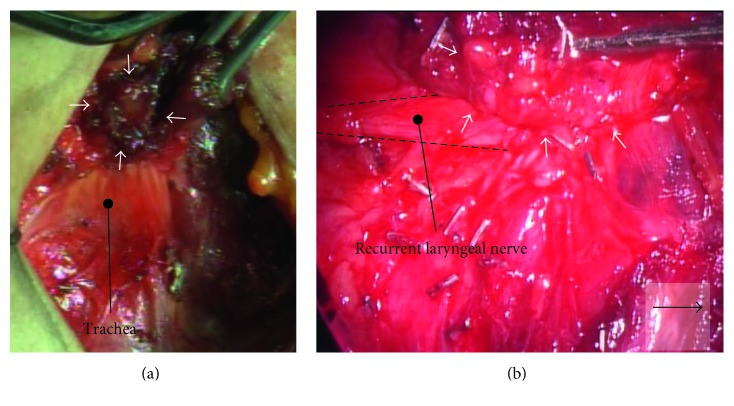
Surgical findings. The left lobe of the thyroid is absent. Tumor is observed on the side of the absent thyroid and has invaded the lateral branch of the recurrent laryngeal nerve. No abnormalities in the path of the recurrent laryngeal nerve are observed. Arrowheads indicate tumor; arrow indicates craniad.

**Figure 3:**
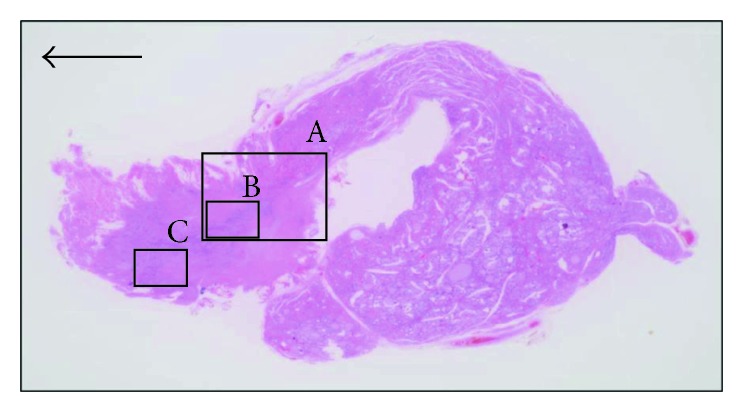
Tumor histopathology. Although the right thyroid lobe and isthmus were evident, left lobe tissue was absent. Most of the tumor represented papillary carcinoma, with a minority portion comprising poorly differentiated carcinoma. No thyroid tissue was observed in the absent portion. Arrow indicates right side. Hematoxylin and eosin staining, ×1.

**Figure 4:**
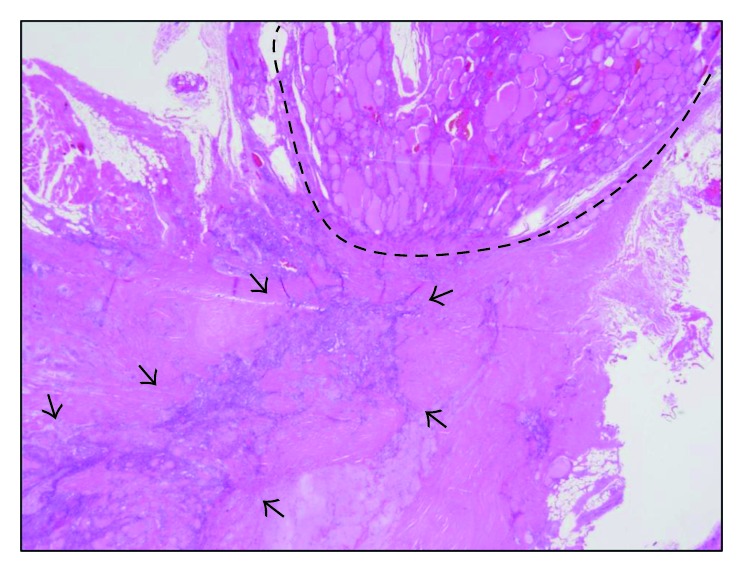
Histopathology of Section A in Figure 3. Clear demarcation is evident between the thyroid tissue and tumor. Dotted line delineates normal thyroid tissue truncating at the isthmus. Arrowheads point to the tumor site. Hematoxylin and eosin staining, ×1.25.

**Figure 5:**
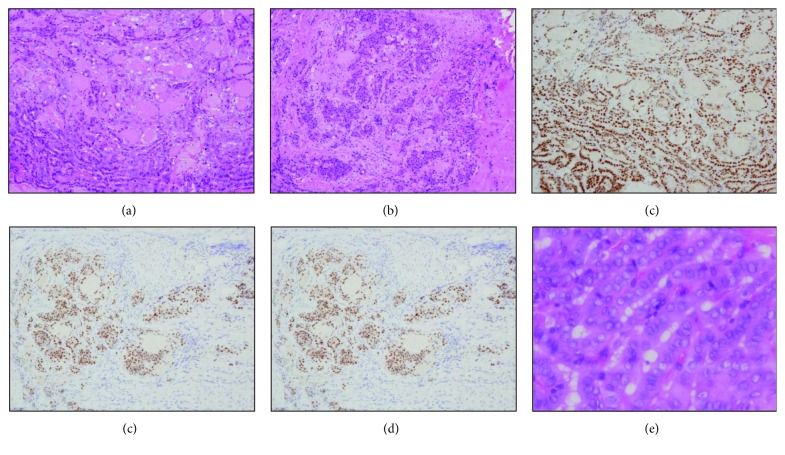
Histopathological (hematoxylin and eosin) and immunohistochemical findings for Sections B and C in Figure 3. Images (a), (c), and (d) are from Section B (thyroid papillary carcinoma), while images (b) and (d) are from Section C (poorly differentiated carcinoma) in Figure 3. (a) The tumor shows a ductal structure with funicular and microfollicular shapes, consistent with thyroid papillary carcinoma. (b) A honeycomb structure and necrosis are observed, with marked differences in nucleus sizes; these findings suggest poorly differentiated carcinoma. (c, d) Both papillary carcinoma and poorly differentiated carcinoma sites are thyroid transcription factor-1-positive. (e) Nuclear grooves under high magnification.

The patient underwent surgery using a standard collar incision. Intraoperative findings confirmed the presence of the right lobe and isthmus, the absence of the left lobe, and the presence of a firm nodule with infiltration into the muscle tissue surrounding the site of the absent lobe on the left of the isthmus after abduction of the anterior cervical muscles. Total right thyroid lobectomy isthmectomy, resection of the tumor, and central neck dissection were performed concurrently. The tumor had invaded the lateral branch of the recurrent laryngeal nerve, which was also resected, but the medial branch of the recurrent nerve was preserved (Figure 2). On the side of the absent thyroid, the superior thyroid artery, superior thyroid vein, middle thyroid artery, inferior thyroid artery, inferior thyroid vein, and parathyroid glands were in the normal locations. No abnormality was evident in the pathway of the recurrent laryngeal nerve. Postoperative histopathological examination found that the right lobe and isthmus were present and the left lobe was absent. The tumor was adjacent to the left side of the isthmus (Figure 3), and a clear demarcation was evident between the thyroid and tumor (Figure 4). Most of the tumor was consistent with thyroid papillary carcinoma with a funicular- and microfollicular-shaped ductal structure, and nuclear grooves were observed within the tumor cells at high magnification (Figures 5 and 5). A honeycomb structure and necrosis were observed in a section of the infiltrating tumor, and a marked difference was seen in the sizes of nuclei, indicative of poorly differentiated thyroid carcinoma (Figure 5). In addition, immunohistochemical staining showed that both the papillary and poorly differentiated carcinomas were thyroid transcription factor- (TTF-) 1-positive, suggesting that the carcinomas originated from thyroid tissue (Figures 5 and 5). The final diagnosis was concomitant papillary thyroid carcinoma and poorly differentiated carcinoma pT4pN0pEx2, with recurrent laryngeal nerve invasion. At 3 months postoperatively, the patient underwent ^131^I ablation on an outpatient basis. Subsequent thyroid scintigraphy indicated no accumulation of radioactive iodine. The patient has remained recurrence-free as of 3 years postoperatively, and observation is continuing.

## 3. Discussion

Thyroid hemiagenesis was first reported by Henle in 1866 [3]. The thyroid develops via epithelial cell proliferation from the foramen cecum of the tongue between the tuberculum impar and copula linguae in week 5 of gestation. The gland subsequently descends as a diverticulum dividing into right and left lobes, reaching the anterior of the trachea in week 7 of gestation [4]. Thyroid hemiagenesis is postulated to be caused by failure of the diverticulum to divide, but some reports have also described associations between a lingual thyroid and hemiagenesis [5, 6]. Confirmation that other locations remain free of abnormal accumulation on thyroid scintigraphy is therefore necessary, even if thyroid hemiagenesis is observed on ultrasonography, CT, or other modalities. In this case, thyroid scintigraphy was not performed before surgery. However, no abnormal accumulation was detected at other sites on thyroid scintigraphy performed postoperatively following ^131^I ablation.

Only 13 cases of thyroid cancer complicated by thyroid hemiagenesis have been reported [7–18]. These cases included 11 female and two male patients, ranging between 14 and 74 years in age. In eight of these 13 cases, the tumors were discovered by palpation following cervical swelling or nodule detection. Five cases, including ours, were asymptomatic. Papillary carcinoma was the most common histology in 10 cases, with one case of follicular carcinoma, one of medullary carcinoma, and one of combined papillary and follicular carcinoma. Our case showed a combination of papillary and poorly differentiated carcinomas, representing the first case in which poorly differentiated carcinoma was identified. Among cases reported to date, the left side of the thyroid was absent in eight patients, and the right side was absent in five. Tumors in all cases except ours arose in the existing parts of the thyroid (i.e., in the lobe or isthmus). In our case, however, the tumor arose on the side of the absent thyroid. The fact that positive results for TTF-1 were obtained for both the papillary and poorly differentiated carcinoma sites suggested that the cancer originated in the thyroid. However, checking for thyroid tissues on the side of the absent thyroid was not possible, so the origin of the thyroid cancer in our patient could not be confirmed.

**Figure 6:**
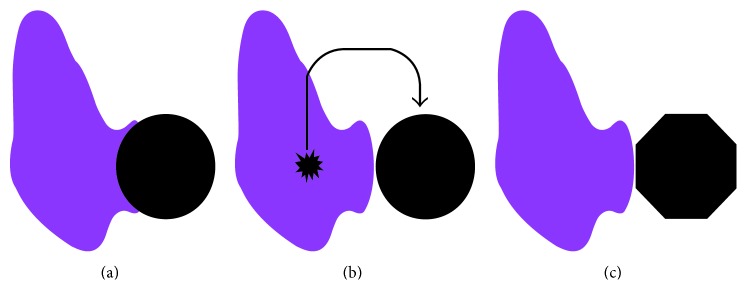
Three hypotheses for tumorigenesis in the present case. (a) Hypoplasia of the left thyroid lobe plus thyroid cancer occurring on the hypoplastic side, with tumor replacing the hypoplastic tissue. (b) Thyroid hemiagenesis plus thyroid microcancer plus lymph node metastasis. (c) Thyroid hemiagenesis plus ectopic thyroid on the side of the absent gland plus ectopic thyroid carcinoma.

We formulated three hypotheses regarding thyroid carcinogenesis in our patient (Figure 6), as outlined below.

*Theory 1.* Hypoplasia of the left thyroid lobe plus thyroid cancer arising on the hypoplastic side.

This hypothesis was ruled out, as the cancer was not contiguous with thyroid gland tissue on histopathological examination.

*Theory 2.* Thyroid hemiagenesis plus thyroid microcancer plus lymph node metastasis.

An extremely small primary lesion could have been present within thyroid tissue in the right lobe, with further lymph node metastasis to the site of the absent gland on the left resulting in the lesion. Multiple reports have described thyroid microcancer and lymph node metastasis to date, as reviewed by Anastasilakis et al. [19] Even a primary as small as 2 mm can lead to cervical lymph node metastasis. In our case, pathological examination of the residual thyroid using 5 mm thick slices found no microcancer. In addition, we were unable to confirm the existence of lymph tissue on the tumor side. This hypothesis thus does not seem to be supported.

*Theory 3.* Thyroid hemiagenesis plus ectopic thyroid carcinoma.

Ectopic noncontiguous thyroid could have been present with residual thyroid on the absent side, where the cancer originated and eventually replaced the ectopic thyroid. This is very rare, as only five cases have been reported in which thyroid hemiagenesis was complicated by an ectopic thyroid [5, 6, 13, 20, 21], and none involved cancer. In addition, ectopic thyroids are mainly observed originating from the tongue, in front of the larynx, or (rarely) at the paratrachea, esophagus, or within the mediastinum[22]. In the present case, the cancer could have developed in an ectopic thyroid arising paratracheally following thyroid hemiagenesis. However, the lack of thyroid tissue on the tumor side makes this hypothesis difficult to substantiate.

Although we consider Hypothesis 3 as the most likely, the mechanism of onset in this case of thyroid carcinoma on the side of the absent thyroid cannot be confirmed.

In summary, this case report appears to represent the first to describe thyroid hemiagenesis complicated by thyroid carcinoma occurring on the side of the absent thyroid and is therefore extremely rare.
